# Epidemiology of Suicidal Behavior in Malaga (Spain): An Approach From the Prehospital Emergency Service

**DOI:** 10.3389/fpsyt.2019.00111

**Published:** 2019-03-13

**Authors:** Berta Moreno-Küstner, José del Campo-Ávila, Ana Ruíz-Ibáñez, Ana I. Martínez-García, Serafina Castro-Zamudio, Gonzalo Ramos-Jiménez, José Guzmán-Parra

**Affiliations:** ^1^Departamento de Personalidad, Evaluación y Tratamiento Psicológico, Universidad de Málaga, Málaga, Spain; ^2^Grupo Andaluz de Investigación Psicosocial (GAP) (CTS-945), Instituto de Biomedicina de Málaga (IBIMA), Málaga, Spain; ^3^Departamento de Lenguajes y Ciencias de la Computación, Universidad de Málaga, Málaga, Spain; ^4^Unidad de Gestión Clínica del Dispositivo de Cuidados Críticos y Urgencias del Distrito Sanitario Málaga-Coín-Guadalhorce, Málaga, Spain; ^5^Unidad de Salud Mental del Hospital Regional Universitario de Málaga, Málaga, Spain

**Keywords:** prehospital emergency services, suicide, suicide attempt, epidemiology, suicidal behavior, risk factors

## Abstract

**Objective:** This study aims to analyse the number and characteristics of calls made to the Málaga Prehospital Emergency Service (PES) for suicidal behavior based on sociodemographic, temporal, and health care variables.

**Method:** This is a retrospective, descriptive study that records all calls made to the PES due to suicidal behavior (suicide attempts and completed suicides) in 2014. Sociodemographic variables (age, sex, and health district), variables related to the calls (time-slot, degree of sunlight, type of day, month, season of the year, prioritization, and number of resources mobilized) were extracted from these calls. The number of cases and percentages were presented for the qualitative variables. The rates per 100,000 were calculated by sex and health district and presented with the corresponding 95% confidence interval (CI).

**Results:** Of the total valid calls to PES (*n* = 181,824), 1,728 calls were made due to suicidal behavior (0.9%). The mean age was 43.21 (±18) years, 57.4% were women, and the rate was 112.1 per 100,000 inhabitants. The calls due to suicidal behavior were in the younger-middle age segment, in the time-slot between 16 and 23 h and during daylight hours, on bank holidays, in spring and summer in comparison with winter, and with a peak of calls in August. The majority of these calls were classified as undelayable emergencies and mobilized one health resource.

**Conclusions:** Prehospital emergency services are the first contact to the sanitary services of persons or families with suicide attempts. This information should be a priority to offer a complete overview of the suicide behavior.

## Introduction

The World Health Organization (WHO) recognizes suicide as a public health priority (1). Approximately 800,000 people commit suicide worldwide each year, resulting in an overall mortality rate of 16 per 100,000 inhabitants and with the number of suicides in an ever-increasing rise (2). In 2014, the suicide rate was 11.25 per 100,000 inhabitants in Europe (3) and 9.5 per 100,000 inhabitants in Spain (4). Suicide is the leading external cause of death in men (5). The southern Spanish region of Andalusia also recorded a suicide rate of 9.34 per 100,000 inhabitants in 2014 (6).

However, these figures do not include suicide attempts that did not actually result in death. A previous suicide attempt is the most important and predictive risk factor for suicide as indicated by Bostwick et al. (7), who estimated that people with a history of self-injury were 25 times more likely to commit suicide than others. Moreover, it has been estimated that for every completed suicide, there are 20 previous attempts, so identifying and following up on these events should be critical for suicide prevention (2). In Andalusia (Spain), suicide attempts have increased following the economic recession that began in 2008 (8).

Considering the importance of identifying suicide attempts and related behavior, more studies should be conducted in the health services, specifically in emergency services, both in hospital and prehospital services, as these are the first places where the person arrives, and much information can be collected on suicide attempts and related behavior. Hence, there is increased importance acquired by pre-hospital emergencies. In an emergency service in the Spanish region of Galicia, Vázquez-Lima et al. (9) found that previous suicide attempts were present in almost half of the patients who completed suicide, a finding that coincides with subsequent studies (10, 11).

Given the results from previous studies in the same research area, we consider it of utmost interest to continue analyzing and updating information where differential characteristics were found in the calls made to the Malaga Prehospital Emergency Service (PES) due to suicidal behavior (12, 13).

The present research aims to study the number and characteristics of calls made to the Malaga PES due to suicidal behavior (including threats, attempts, and completed suicide) by reviewing records of the public emergency healthcare database.

## Methods

### Study Design

The research consists of a descriptive, observational, and cross-sectional study of the calls made to the PES due to suicidal behavior in the province of Malaga during 2014. The population of the Malaga province at the time was roughly 1,541,831 inhabitants. The study met the ethical criteria for research and was approved by the Malaga Ethics and Research Committee.

### Sources of Information and Selection of Records

The information recorded in the computerized database of the Malaga Urgencies and Emergency Coordination Centre (UECC), including the calls made to the PES (number 061 or 112) of this province was considered to carry out this study. The procedure followed by the Malaga UECC is that once the telephone call has been made, the operator and unit doctor record all information on the event to choose the best-suited resource (ambulance, helicopter, etc.) according to the reason and priority of the call. If deemed necessary, a medical team will travel to the scene to attend to the person calling for service.

In the process of selecting the cases to be included in this study, the following information collected at three different levels was considered: (1) the type of call according to the UECC classification given by the operator or coordinator doctor at the time of the call, (2) the “Clinical Judgement” offered by the health professionals attending the patient at the scene, and (3) the type of outcome when the PES team arrives at the scene. These three levels are:

1. The classification made by the UECC operator consists of 14 categories (Table 1). Within these, the category called *psychiatric calls* includes elements such as nervousness, incoherence/confusion, opposition, sadness, violence, and anxiety, among others. Calls recorded in this category related to suicidal behavior (such as self-injury and suicidal tendency, suicidal thoughts, suicide threat, and suicide) were included in the suicidal behavior category (Table 1).
2. Concerning information regarding “Clinical Judgement,” the calls whose International Classification of Diseases (ICD-9) codes were related to suicidal behavior were selected. These codes were V62.84, including those referring to suicidal ideation, and codes from E950 to E959, referring to suicide and self-inflicted injuries. These codes are listed in Table 1.
3. Finally, cases where the PES team directly visit the site and the outcome was fatal (*exitus*) were selected.

**Table 1:** Classification of the Urgencies and Emergencies Coordinating Center of Malaga and classification of suicidal behaviors according to the International Classification of Diseases (ICD-9).

**Classification of the urgencies and emergency coordinating center of malaga**	
Non-traumatic pain	
Neurological and/or level of consciousness	
Dyspnoea	
Trauma	
Alteration of vital signs	
Psychiatric illness^*^	
Traffic accidents	
Gastrointestinal	
Nursing calls	
Poisoning/allergies	
Hemorrhages	
Gynecological/obstetric/urinary	
Environmental Emergencies/external agents	
Others	
**Code**	**Definition**
**INTERNATIONAL CLASSIFICATION OF DISEASES, 9^TH^ REVISION**	
V.62.84	Suicide ideation
E950	Suicide and self-inflicted poisoning by solid or liquid substances
E951	Suicide and self-inflicted poisoning by gases in domestic use
E952	Suicide and self-inflicted poisoning by other gases and vapors
E953	Suicide and self-inflicted injury by hanging, strangulation and suffocation
E954	Suicide and self-inflicted injury by submersion (drowning)
E955	Suicide and self-inflicted injury by firearms, air guns and explosives
E956	Suicide and self-inflicted injury by cutting and piercing instrument
E957	Suicide and self-inflicted injury by jumping from high places
E958	Suicide and self-inflicted injury by other and unspecified means
E959	Late effects of self-inflicted injury

* *In this category, calls related to “self-injury and suicidal tendency, suicidal thoughts, suicide threated and suicide were selected for this study*.

### Study Variables

The outcomes of suicidal behavior include self-injury and suicidal tendency, suicidal thoughts, suicide threat, and consummated suicide (or *exitus*).

The variables considered for users were sex (female/male) and age (categorized in intervals: 0–17, 18–29, 30–44, 45–59, 60–75, and 75+). The health district in which the person calling the service lives was also analyzed (Ronda, Antequera, Axarquía, Coín-Guadalhorce, Costa del Sol, or Malaga city).

The temporal variables of the calls were: time-slot (from 0:00 to 7:59, 8:00 to 15:59, or 16:00 to 23:59), degree of sunlight (sunrise or sunset), type of day (working days or bank holidays), month of the year, and seasonal distribution (winter, autumn, spring, or summer).

Finally, health care information was analyzed according to the prioritization of the call, from highest to lowest priority (4: emergency, 3: undelayable emergency, 2: delayable emergency, or 1: not urgent) and the number of resources mobilized (one or more), where the mobilized resource is understood to be the intervention of an ambulance.

### Statistical Analysis

Rates were calculated based on the total population of the province of Málaga and by health district and presented by 100,000 inhabitants. The reference population was provided by the Andalusian Health Service relating to the health cards of 2014 distributed by health district in the province of Malaga. Rates were calculated using the direct method and the confidence interval:

$$\begin{array}{l} t=\frac{n}{N} \end{array}$$

where *t* = gross rate, *n* = number of cases, and *N* = person-years.

The following formula was used to calculate the confidence intervals:

$$\begin{array}{l} t\pm 1.96\sqrt{\frac{t\;\times \left(1-t\right)}{N}} \end{array}$$

where *t* = gross rate and *N* = person-years.

The arithmetic mean and standard deviation (SD) were used to describe the quantitative variables. Qualitative variables were expressed with the number of cases and percentage. For percentages, the confident intervals were built using bootstrapping (1,000 samples). All confident intervals were at 95%. The SPSS statistical package (version 17) and Excel were used in a Windows operating system.

## Results

The analyzed database consisted of 299,405 calls. For this study, calls unrelated to health (those that did not involve assistance to people with health needs; *n* = 56,273) and those not classified in any specific category by the UECC (*n* = 30,198) were eliminated from the sample. The database was also subject to a quality control process in which duplicate records (records that matched the identification number, date, and time) and registry errors (*n* = 31,110) were eliminated from the sample.

**Figure 1:**
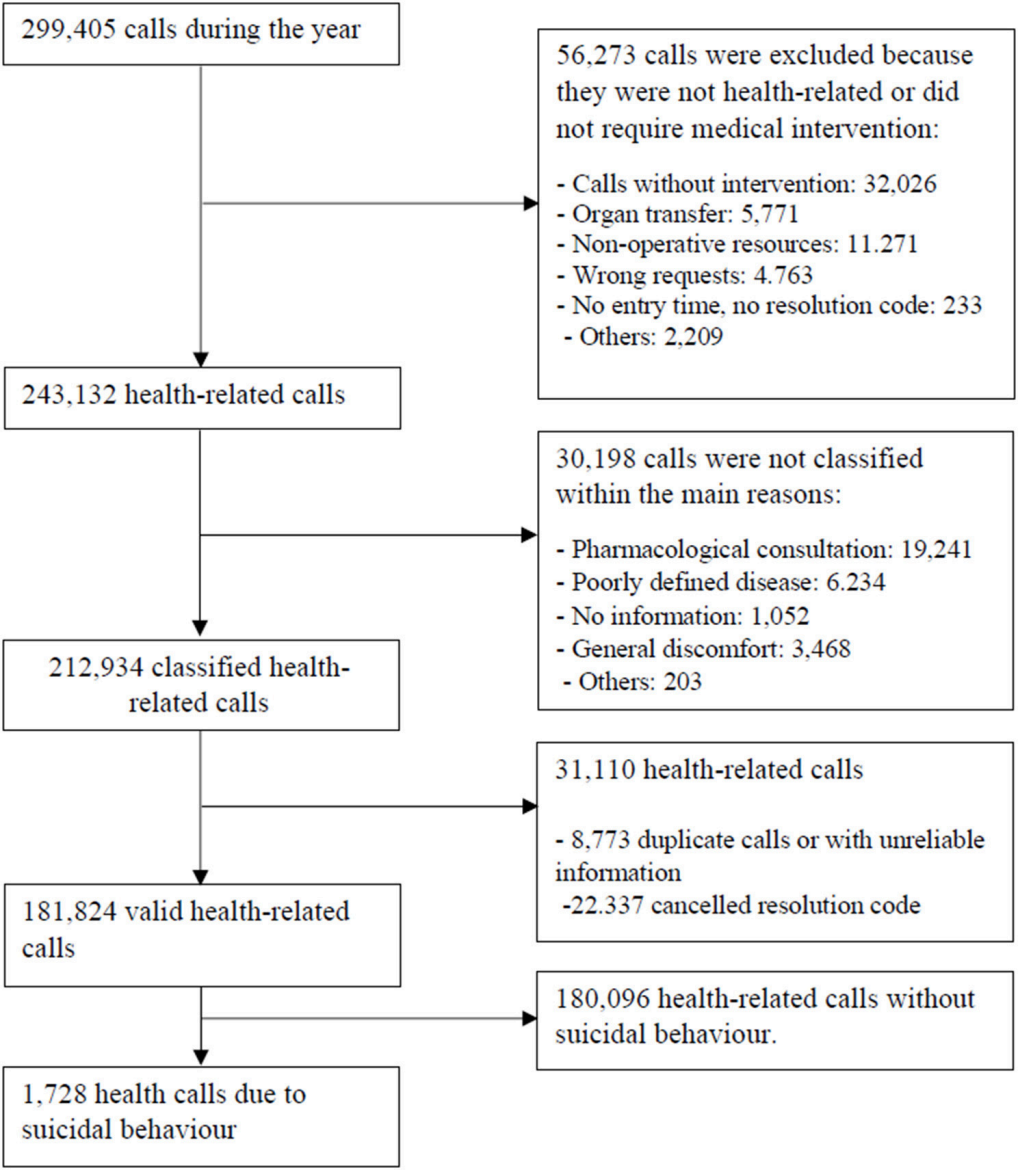
Flow chart of the study sample selection process.

Of the 181,824 valid calls to the PES in the Malaga province, 1,728 (0.9%) were due to suicidal behavior. Further information on the sample selection process is shown in Figure 1.

With respect to sociodemographic variables, the mean age of people with suicidal behavior was 43.21 (*SD* = 18.26). The 30–44 age group proved to make the most calls due to suicidal behavior, followed by the 45–59 group (Table 2). Women made 1.3 times more calls than men (56.5% [95% CI: 54.2 to 58.7] vs. 43.5% [95%CI: 41.3 to 45.8]).

**Table 2:** Distribution of the suicidal behavior calls made to the Prehospital Emergency Service according to age and sex.

**Age groups**	**Total**			**Men**			**Women**		
	***n***	**%**	**CI 95%**	***n***	**%**	**CI 95%**	***n***	**%**	**CI 95%**
<18 years	71	4.3	3.4–5.3	16	2.2	1.3–3.3	55	5.9	4.4–7.4
18–29 years	246	14.9	13.2–16.6	127	17.6	14.9–2.5	119	12.7	10.4–14.8
30–44 years	622	37.6	35.3–39.9	295	40.9	37.4–44.5	327	35.0	31.8–38.2
45–59 years	519	31.3	28.9–33.5	200	27.7	24.6–30.8	319	34.1	31.4–37.0
60–75 years	141	8.6	7.2–9.9	47	6.5	4.7–8.3	94	10.1	8.2–11.9
>75 years	57	3.4	2.6–4.4	37	5.1	3.7–6.8	20	2.1	1.3–3.1
Total	1656	100		722	100		934	100	

*Missing data n = 72*.

The rate of suicidal behavior calls made to the Malaga PES in 2014 was 112.07 per 100,000 inhabitants. Regarding health districts of residence, Malaga city recorded the highest total suicide rate (121.70 per 100,000 inhabitants), and Ronda had the lowest rate (94.62 per 100,000 inhabitants; Table 3). There were no statistically significant differences between the different regions regarding suicide rate. When comparing rates by sex, there was a higher overall rate in women (118.88 per 100,000 inhabitants) compared to men (96.29 per 100,000 inhabitants; *p* < 0.05) with a ratio of 1.2.

**Table 3:** Distribution of the suicidal behavior calls made to the Prehospital Emergency Service by health district.

**Health district**	**Population**	**Total calls**	**Suicidal calls**	**Rate x 100,000**	**CI 95%**	**% of total calls**
Ronda	53,901	8,088	51	94.62	68.66–120.57	0.6
Antequera	109,678	15,001	109	99.38	80.73–118.03	0.7
Axarquía	150,033	22,070	161	107.31	90.74–123.88	0.7
Coín- Guadalhorce	134,944	14,701	144	106.71	89.29–124.13	1.0
Costa del Sol	475,354	46,304	511	107.50	98.18–116.81	1.1
Málaga Capital	617,921	75,520	752	121.70	113.01–130.39	1.0
Total	1,541,831	181,684	1728	112.07	106.79–117.36	0.9

*CI: Confidence Interval*.

Regarding the temporality variables of suicidal behavior calls (Table 4), more calls were made for suicidal behavior in the timeslot between 16:00 and 23:59 (48.1%). Considering the solar calendar, more calls were made at sunrise (57.3%) than at sunset. Regarding the months, there were more calls in the month of August compared to January and February and more in October compared to February (*p* < 0.05). There was a higher percentage of calls in the spring and summer compared to winter (*p* < 0.05).

**Table 4:** Distribution of suicidal behavior calls made to Prehospital Emergency Service by temporal and care variables.

**Variables**	**Suicidal behavior calls**		
	***n***	**%**	**CI 95%**
**TIME SLOT**			
0:00–7:59	307	18.8	16.0–19.6
8:00–15:59	589	34.1	31.9–36.5
16:00–23:59	832	48.1	45.7–50.5
**DEGREE OF SUNLIGHT**			
Sunrise	990	57.3	55.0–59.6
Sunset	738	42.7	40.4–45.0
**MONTHS OF THE YEAR**			
January	123	7.1	5.9–8.4
February	112	6.5	5.4–7.7
March	151	8.7	7.5–10.1
April	131	7.6	6.4–9.0
May	152	8.8	7.6–10.2
June	148	8.6	7.2–9.9
July	152	8.8	7.3–10.1
August	172	10.0	8.5–11.3
September	156	9.0	7.7–10.4
October	167	9.7	8.3–11.2
November	125	7.2	6.0–8.4
December	139	8.0	6.8–9.4
**SEASONS**			
Winter	375	21.7	19.7–23.6
Spring	445	25.8	23.7–27.7
Summer	474	27.4	25.3–29.5
Autumn	434	25.1	23.0–27.1
**PRIORITY**			
Emergencies	148	8.6	7.3–9.8
Non-deferrable emergencies	1539	89.1	87.6–90.5
Deferrable emergencies	35	2.0	1.3–2.7
Not urgent	6	0.3	0.1–0.6
**NO OF RESOURCES MOBILIZED**			
One	1682	97.3	96.6–98.1
More than one	46	2.7	1.9–3.4
**TYPE OF DAY**			
Working day	1163	67.3 (3.87^*^)	64.8–69.3
Bank holiday	565	32.7 (8.69^*^)	30.7–35.2

* *Mean calls per day based on 65 bank holidays and 300 working days during 2014*.

The mean number of calls due to suicidal behavior on bank holidays and working days was calculated based on the number of calls in each case. There were 300 working days and 65 bank holidays in 2014, and the average number of calls on bank holidays was 8.69, but on working days it was 3.87.

Finally, regarding care information about the functioning of PES, it was noted that 89.1% of calls were classified as undelayable emergencies (priority level 3), while 0.3% were classified as not urgent (priority level 1). In terms of the number of resources mobilized (ambulances), 97% used one resource and 3% used more than one resource.

## Discussion

The main result of this study is that calls made to the PES due to suicidal behavior in the province of Malaga accounted for 0.9% of all calls (*n* = 1,728) and presented a suicide behavior rate of 112.07 per 100,000 inhabitants. Our figure is higher (0.8%) than that reported by Jiménez-Hernández et al. (13) in the same area in 2008. A possible explanation for the increase in this figure is that in the Jiménez-Hernández et al. study, they selected only calls classified by the operator as suicidal behavior, while in our study, we also selected the calls classified by the sanitary staff who attended the caller *in situ* and indicated a clinical judgement of ICD-9 code of suicidal behavior. Comparing our results with studies that used information from PES, the suicide behavior rate we obtained (112.07 per 100,000 inhabitants) in our region was higher than those found in other parts of Spain [76.1 per 100,000 inhabitants found by Vázquez-Lima et al. (9)]. A recent study developed by Mejías et al. (14) offered a figure of 34.7 per 100,000 inhabitants in the whole region of Andalusia, and it presented the highest rate in the province of Malaga (60.0 per 100,000 inhabitants). An explanation for our high results, which are nearly double those found by Mejías et al. (14), is that they have included only calls automatically labeled with the code X84 (intentional self-harm by unspecified means) of the ICD-10, while this code was not used automatically in Malaga in 2014, so we included all calls with terms related to suicidal behavior such as self-injury, suicidal tendency, suicidal thoughts, and suicide threat. This comparison must be made with caution and awareness that we were very inclusive in order to detect as many cases of suicide attempt as possible. However, in USA a study found a rate between 163.1 and 173.8 per 100,000 (15) which were higher in comparison with the results of this study.

Suicidal behavior in our study was higher in women (56.5%) than in men. The same trend was also found in other studies in the literature in which women used emergency services more frequently due to suicidal behavior or suicidal ideation (9, 14, 16, 17). The average age of our sample (43.2 years) is similar to previous studies (14–16) but higher than that of Vázquez-Lima et al. (9). In line with previous studies (12, 13), differences according to age were found with more suicide calls being made by people between the ages of 30 and 44 and between 45 and 59.

Regarding the time interval, our study shows an increase in suicide attempts in the time slot between 16:00 and 23:59, which is in accordance with other studies (14, 16). Doganay et al. (18) also found that suicide attempts were more frequent between 18:00 and 21:00 in men and between 15:00 and 18:00 in women,. Incidentally, we also found an increase in suicidal calls at sunrise (57.3%).

We further observed that there were proportionally more calls on bank holidays than on working days, as noted by Mejías et al. (14). However, there is an inconsistency of results in most of the publications in terms of distribution by months and of the year (9, 13, 16), and homogeneous behavior has not been found. Our results offer a higher frequency of suicide behavior calls during the summer, as observed in previous studies (14, 15, 19, 20). Our findings corroborate the temporal distribution of the current body of knowledge in the field.

As for the priority level, a high percentage of calls were classified as urgent and non-deferrable (89.1%), which is to say priority level 3 (the maximum being level 4), due to the severity of these calls and the danger of the emergency being fatal in accordance with figures reported by Mejías et al. (14) for all of Andalusia. Finally, a higher proportion of calls mobilizing one health resource was found with its implicit economic cost. This result was similar to that of Jiménez-Hernández et al. (13), who asserted that most suicidal behavior emergencies entailed a high economic cost because they mobilized one ambulance.

In conclusion, the results suggest that people who made suicidal behavior calls to the PES in Malaga were in the younger-middle age segment, more frequently women, both sexes called more frequently in the time-slot between 16:00 and 23:59 and during the daylight hours, there were more calls due to suicidal behavior on bank holidays than working days, and there were more calls in spring and summer than in winter with a peak of calls in August. In addition, the clear majority of these calls were classified as priority level 3 (non-deferrable emergencies) and frequently mobilized one health resource.

### Limitations

Several limitations must be highlighted in this study. The most significant limitation relates to the problems arising from the cross-sectional study design. A second limitation is the scarcity of validated clinical information recorded by emergency health personnel, as there is a possibility that some suicide attempts might not have been included in the sample due to coding errors on the part of the operator. An example could be cataloging a call as drug intoxication even though the person might actually be attempting suicide. In this case, this call would not have been included in the analysis. However, although this is a considerable limitation, the study also has the advantage that the data analyzed were collected from daily clinical practice in the prehospital emergency service and correspond to all calls made in the province of Malaga. It can, therefore, be considered that these data accurately represent the calls made to the PES in this province.

Another significant limitation of our study has to do with the Malaga UECC's classification system, which does not follow an international classification method, and there are no homogeneous registration methods for suicide attempts. Therefore, the data obtained should be compared with caution with those of other communities or international studies. Regarding the comparison between different regions of Malaga, the small populations compared increase the probability of type II errors. Finally, inter-rater reliability between UECC operators is not reported and could be an important issue for estimating the rate of behavior suicide calls. However, one of our main strengths is that this study is closer to the real number of calls made to the PES due to suicidal behavior.

## Conclusion

Research studies based on pre-hospital clinical databases are very scarce. As pre-hospital services are the first contact to the sanitary services of persons or families with suicidal behavior, this information should be a priority in order to offer a complete overview of the suicide behavior, as it is closely related to suicide completion. As Málaga presents a higher rate of suicide attempts compared with other parts of Spain, further investigations are needed in this province in order to find possible explanations for these findings.

## Author Contributions

BM-K and JG-P were involved in the conception, design, interpretation of data, and drafting the article. JdC-A and GR-J were involved in the design, analysis, interpretation of data, and drafting the article. AR-I, AM-G, and SC-Z were involved in the interpretation of data and drafting the article. All authors provided final approval of the version to be published.

### Conflict of Interest Statement

The authors declare that the research was conducted in the absence of any commercial or financial relationships that could be construed as a potential conflict of interest.
